# Inflammatory Diseases Among Norwegian LRRK2 Mutation Carriers. A 15-Years Follow-Up of a Cohort

**DOI:** 10.3389/fnins.2021.634666

**Published:** 2021-01-28

**Authors:** Jan O. Aasly

**Affiliations:** ^1^Department of Neurology, St. Olavs Hospital, Trondheim, Norway; ^2^Department of Neuromedicine and Movement Science (INB), Faculty of Medicine and Health Sciences, Norwegian University of Science and Technology (NTNU), Trondheim, Norway

**Keywords:** Parkinson’s disease, LRRK2, inflammation, multiple sclerosis, rheumatoid arthritis, achalasia, dementia

## Abstract

The first families with LRRK2 related Parkinson’s disease (PD) were presented around 15 years ago and numerous papers have described the characteristics of the *LRRK2* phenotype. The prevalence of autosomal dominant PD varies around the world mainly depending on local founder effects. The highest prevalence of *LRRK2* G2019S PD in Norway is located to the central part of the country and most families could be traced back to common ancestors. The typical Norwegian LRRK2 phenotype is not different from classical PD and similar to that seen in most other LRRK2 families. The discovery of LRRK2 PD has allowed us to follow-up multi-incident families and to study their phenotype longitudinally. In the Norwegian LRRK2 families there has been a significantly higher incidence of inflammatory diseases like multiple sclerosis and rheumatoid arthritis that seen in other PD populations. Recent studies in LRRK2 mechanisms have indicated that this protein may be crucial in initiating disease processes. In this short survey of 100 Norwegian mutation carriers followed through more than 15 years are presented. The prevalence of inflammatory diseases among these cases is highlighted. The role of LRRK2 in the conversion process from carrier status to PD phenotype is still unknown and disease generating mechanisms important for initiating LRRK2 PD are still to be identified.

## Introduction

The etiology of Parkinson’s disease, PD, is unknown and for many years it was regarded as a sporadic disease explained by environmental causes. The first PD gene locus was reported in 1996 and 1 year later the gene was located to the *SNCA* coding for α-synuclein (Polymeropoulos et al., 1997). This protein was later shown to constitute a major part of the Lewy-bodies, the pathoanatomical hallmark of Parkinson’s disease (Spillantini et al., 1997). During the following years three important genes coding for autosomal recessive PD were found, one for *parkin*, *PRKN*, the most common gene for young onset parkinsonism, and *PINK*1 and *DJ-1* as autosomal recessive causes of PD (Kitada et al., 1998; Valente et al., 2001; Bonifati et al., 2003). A Japanese group had pointed to an important locus on chromosome 12 and mutations in the *LRRK2* gene were finally identified by several other groups in 2004. *LRRK2* is probably the most common cause of autosomal dominant PD and the most common monogenic form of PD (Paisan-Ruiz et al., 2004; Zimprich et al., 2004).

The discovery of *LRRK2* as a major cause of PD has led to a tremendous race of new biomarkers for PD and new insights on disease pathogenesis (Paisan-Ruiz et al., 2004). The *LRRK2 G2019S* is the most prevalent risk factor among the *LRRK2* mutations. It is most common in the Middle East and there is a very clear south north gradient in distribution. The prevalence of *LRRK2* G2019S PD in Scandinavia is low with an exception located to the northwestern coast of Norway. This cohort of PD patients has been followed for many years and were included in many studies for better understanding of clinical and biochemical processes related to PD (Aasly et al., 2005). Although the *LRRK2* PD phenotype is rather close to classical PD the long-term follow up of Norwegian LRRK2 cases have shown that inflammatory mechanism may contribute to the disease process.

Neuroinflammation is now considered to play a major role in the pathogenesis of PD. This change in paradigm has come after findings of activated microglia and upregulation of cytokines in PD brains. One of the most important substances seem to be cyclooxygenase-1 and -2, which show increased expression, together with inflammatory cytokines and inflammatory-related substances. The risk for PD is correlated with inflammatory cytokine genes (i.e., tumor necrosis factor-α and interleukin-1β) polymorphisms and with cell-surface human leukocyte antigen2 (Crotty et al., 2020).

The role of the immune system and inflammation and LRRK2 upregulation is also being increasingly explored and coupled to the innate and adaptive immune system. Large multi-center whole exome, WES, -studies have shown that the *LRRK2* gene is associated with several chronic inflammatory disorders, including Crohn’s disease and leprosy (Hakimi et al., 2011).

The aims of this survey are to present the long-time follow up of the original Norwegian LRRK2 cohort and to discuss the possible importance of recognizing the high prevalence of inflammatory diseases among these cases.

### Clinical Material

The *LRRK2* G2019S cohort in central Norway was first presented in Aasly et al. (2005). The families had been followed for several years and more subjects were added to the material within a few years after the discovery of the mutation. The patients were identified and included as part of a screening program of multi-incident PD families living at the coastline of central Norway. When the *LRRK2* gene was found and connected to PD, these families were further evaluated and characterized.

In this survey of the first 100 Norwegians known to carry the a heterozygous *LRRK2* G2019S mutation, are presented. Twenty-nine patients out of the first 100 cases had developed PD at the time when they were identified through the family screening program. Three more cases converted to PD through the follow-up period. The age of onset of the 32 first PD cases varied substantially, with an average onset of 61.2 years. The majority of cases had converted to PD in their seventh decade. Nine cases, 29%, were in their sixties at disease onset and the mean age at onset and for this small group the mean onset was 64 years. Two patients had disease onset in their late thirties and two cases showed first signs of tremor and bradykinesia up in their eighties (Table 1). The phenotypical features at onset were similar to that commonly found in sporadic PD. All cases had asymmetric signs and symptoms, more than 80% showed rest tremor at onset. All patients diagnosed with PD had well preserved cognition at disease onset. The remaining 68 mutation carriers who had not developed PD and were diagnosed as healthy LRRK2 carriers were all assessed as cognitively well-doing. During the follow-up period three asymptomatic LRRK2 mutation carriers developed dementia of Lewy body type, DLB, without typical PD signs. The mean age of the mutation carriers who did not developed PD was 62 years (range 45–83 years) at the end of the follow-up period. The majority of cases had been evaluated clinically several times and had been to repeated PET- or Datscans.

**TABLE 1:** List of 100 LRRK2 G2019S mutation carriers, 15 years-follow-up.

	**Age < 40 years**	**Age 40–49**	**Age 50–59**	**Age 60–69**	**Age 70–79**	**Age 80 –**
No patients Mean onset disease	2/f:1 m:1 38 years	5/f:2 m:3 45 years	8/f:4 m:4 54 years	9/f: 4 m:5 64 years	6/f:4 m:2 75 years	2/f:2 83 years
Healthy carriers	0	11	18	21	15	3
Total number PD/carriers	2	16	26	30	21	5

*Age at onset, AAO, and age at last visit.*

#### Familial Clustering

**FIGURE 1:**
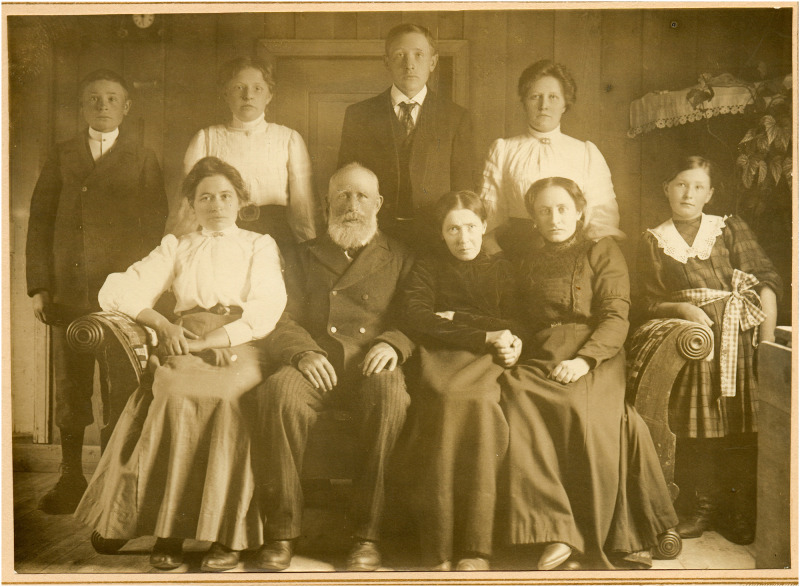
First identified Norwegian LRRK2 patients, family photo 1911 (with permissions from the family).

The vast majority of Norwegian *LRRK2* G2019S mutation carriers known so far, all originate from small settlements along the coastline of Central Norway. The *LRRK2* mutation was found in about 15 families or family branches. The first married couple mentioned by name common to all these families, were born around year 1580 (Johansen et al., 2010). Although this is an autosomal dominant disease, multi-incident PD families were only reported in among half of the cases. In some families with clear familiar clustering of cases this knowledge was more or less kept as a secret and was not a topic for open discussions. When unveiling the cause of the disease these families became very motivated for further collaboration in the struggle for finding new therapeutic remedies. All families had been living in the same area since many generations and most families had families that could give precise information on their ancestors through the last century. One fourth of the families had 3 or more affected family members. Our first identified family LRRK2 PD case with tremor and parkinsonism was born at the time when Charcot named the disease. She had developed the disease in her early forties and had to be taken care of by her family for many years. In a family photo from 1911 the very typical parkinsonian features in her face and body are visualized and even the hand tremor is depicted in the grip of one of her daughters (Figure 1).

### Clinical Phenotypes

About a third of the mutation carriers had developed PD at the time when the follow-up period started 15 years ago. Tremor was the initial parkinsonian sign that brought 3 out of four to the neurologists. This percentage of tremor is well in line with reports from that observed in sporadic PD. The last one-fourth of the cases experienced akinesia, gait problems, micrographia or dystonia as initial signs. This does not differ from other PD populations. Many studies have shown that LRRK2 PD usually respond very well to levodopa and to dopamine agonists. The same was seen in Norwegian cases. Four out of the 32 cases have also ended up with severe complications and needed advanced therapy. In all four patients were treated with deep brain stimulation, DBS, and all four had good or excellent effects. Their mean age at PD onset was 46 years (range 39–59). The first one had DBS 19 years ago at age 49 years. She is still in H&Y III, cognitively intact and living in her own home. It is not well understood why LRRK2 patients with mutations in the kinase domain respond very well to DBS (Schupbach et al., 2007; Johansen et al., 2011; Angeli et al., 2013). This impression is based on reports from several centers around the world. It may also be true for those with mutations in the cor domain, while patients with roc domain mutations seem to have a less favorable outcome from DBS (Gomez-Esteban et al., 2008; Hatano et al., 2014). Some reports on LRRK2 families have noticed that atypical phenotypes also occur (Wszolek et al., 2004). There were no multiple system atrophy or other atypical PD phenotypes observed among members of our families who converted to PD. There was one members diagnosed with MSA observed in one of our families but eventually he tested negative for the G2019S mutation.

### Inflammatory Diseases in LRRK2 G2019S Mutation Carriers Before and After Converting to PD

#### Multiple Sclerosis, MS

Three out of 100 *LRRK2* G2019S mutation carriers were diagnosed with MS. Two cases had no parkinsonian signs or symptoms. The third had mild rigidity and bradykinesia.

Case 1: A 69 years old man, his father had PD. He worked as a carpenter and was a moderate smoker. At the age of 45 year he was diagnosed with retrobulbar neuritis. Two years later he suffered a mild central paresis of the right lower limb with increased tendon reflexes and his right sided plantar reflex was clearly abnormal. Lumbar puncture showed increased cerebrospinal fluid, CSF, immunoglobulins with 5–6 bands not found in his serum. Repeated cerebral MRI scans showed typical MS lesions. His disease was quite benign with little progression. He was not on any medication. At age 69, 24 years after first attack, he was still ambulating and had only minor autonomic dysfunctions; his EDSS grade was 2. He had been to regular follow-up as an asymptomatic LRRK2 carrier and he has developed mild rigidity and bradykinesia but no tremor. A Datscan showed mild reduction of uptake in the putamen bilaterally but not in the caudate nuclei. No anti-parkinsonian therapy was needed.

Case 2: A 57 years old woman was diagnosed with MS at the age of 35 years. CSF bands indicated intrathecal synthesis of immunoglobulins and brain MRI demonstrated periventricular high-signal intensity lesions with typical distribution for multiple sclerosis. Initially she suffered 5–6 attacks and later her disease turned to a more chronic progressive pattern. She was on interferon-beta treatment over a period of 5 years. She had moderate autonomic dysfunctions. Her dominating MS-pattern through all these years has been an extreme feeling of fatigue. Twenty-two years after onset she was still ambulating, living in her own apartment and her EDSS grade was 4. She had moderate spasticity and no rigidity. There was no bradykinesia unrelated to MS. A Datscan was not done. In contrast to the male MS patients she had always been a non-smoker.

Case 3: A 59 years old man, his mother developed PD around the age of 60 years and he is the brother of case 2. There was no additional family history of MS. From age 32 years he suffered multiple attacks of optic neuritis and central nervous manifestations. CSF and MRI examinations showed typical MS pathology. He had a rapid disease progression and reached EDSS grade 4 already after 4 years of disease duration. He stopped smoking around age 50, mainly because of his physical condition. At the age of 59 he was non-ambulating, almost quadriplegic with a tiny rest function of motility in one hand. He had no rigidity and no tremor. His speech was unremarkable but talking could trigger attacks of severe trigeminal neuralgia. His cognitive functions were considered as good. His autonomic dysfunctions were affected and regarded as part of his long-lasting MS. At last examination, age 59 years, his EDSS was between 8.0 and 9. He died from an acute abdominal disease 6 months later. At autopsy his brain weight was 1,290 g with focal demyelinated plaques and variable degrees of inflammation, gliosis, and neurodegeneration. There was no neuron loss in the substantia nigra and multiple stains for tau, amyloid and alpha-synuclein pathology were negative.

### Achalasia

**FIGURE 2:**
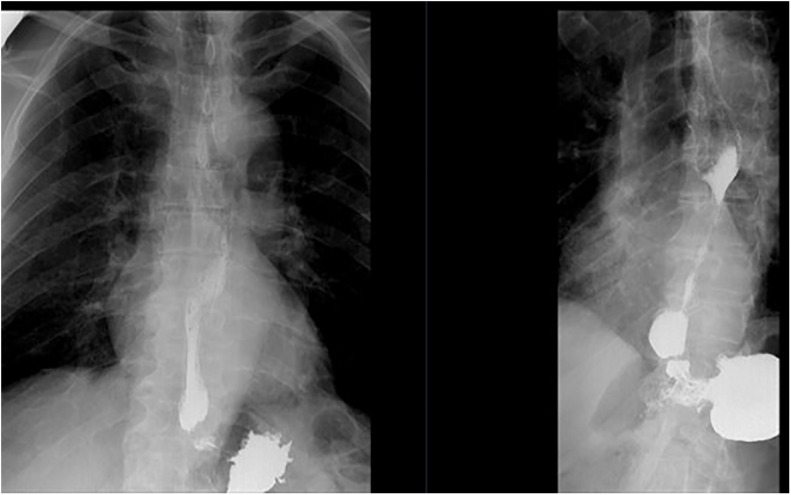
Case 4, achalasia, a barium esophagogram showing a narrowed part of the lower part of the esophagus.

**FIGURE 3:**
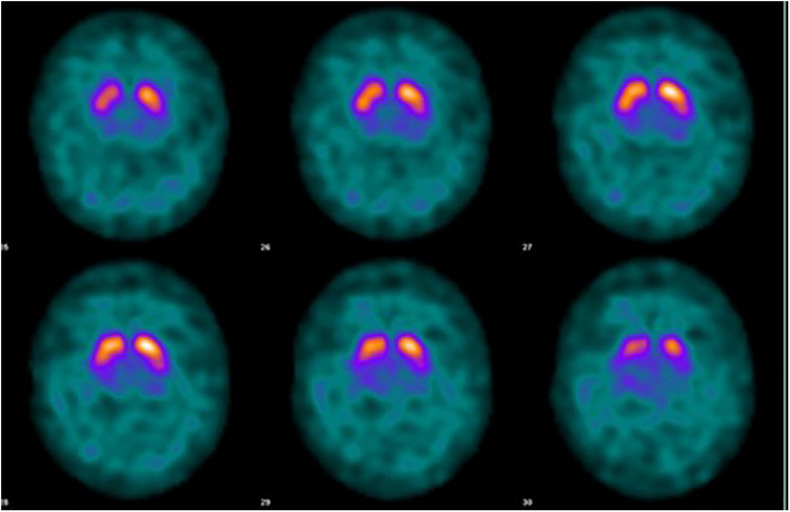
Case 4, Datscan at age 67 years. Bradykinesia and rigidity but no tremor.

Case 4: A 52 years old previously healthy teacher presented with swallowing problems. His mother had PD and was genotyped with the *LRRK2* G2019S mutation. He was on anti-hypertensives and he was a heavy smoker. His dysphagia progressed over the period of some months and he underwent a barium esophagogram which showed a narrowed part of the lower sphincter of the esophagus (Figure 2). It was supplied with high resolution manometry pressure topography, confirming the diagnosis of achalasia. A pneumatic balloon dilatation was successfully performed. Followed-up visits through 18 years have been unremarkable and he had no swallowing problems. No tests for viral agents or immunological causes were performed. At age 54 he tested positive for the G2019S mutation and was included in the long-term follow-up study. During the last 10 years he has developed mild bradykinesia and rigidity but no tremor. A Datscan showed mainly left-sided abnormalities (Figure 3). He does not need anti-parkinsonian therapy. At age 65 he had an acute episode with a ruptured colon diverticulitis. A biopsy from the lower colon did not show any Lewy bodies. His esophagus problems have not recurred and a control esophagogram was unremarkable.

### Rheumatoid Arthritis

Case 5: A 44 years old nursing home assistant was member of a multi-incident PD family and had been followed as a healthy mutation carrier of a heterozygous *LRRK2* G2019S mutation. She was a moderate smoker. Her mother had PD and case 5 was positive for the same mutation. Five years after inclusion she converted to PD. Her most prominent signs were asymmetric rest tremor, bradykinesia and rigidity. She responded well to levodopa and dopamine agonists. In the years before converting to PD she complained of joint stiffness and pain. A diagnostic procedure was performed at the department of rheumatology and their conclusion was sero-positive rheumatoid arthritis. She was treated with local injections and systemic therapy according to current guidelines. Her rheumatic disease progressed and was the main reason for her retirement a few years later. Around the age of 60 years her PD is fluctuating and is in an advanced stage and she has been under consideration for DBS therapy or apomorphine infusions.

**FIGURE 4:**
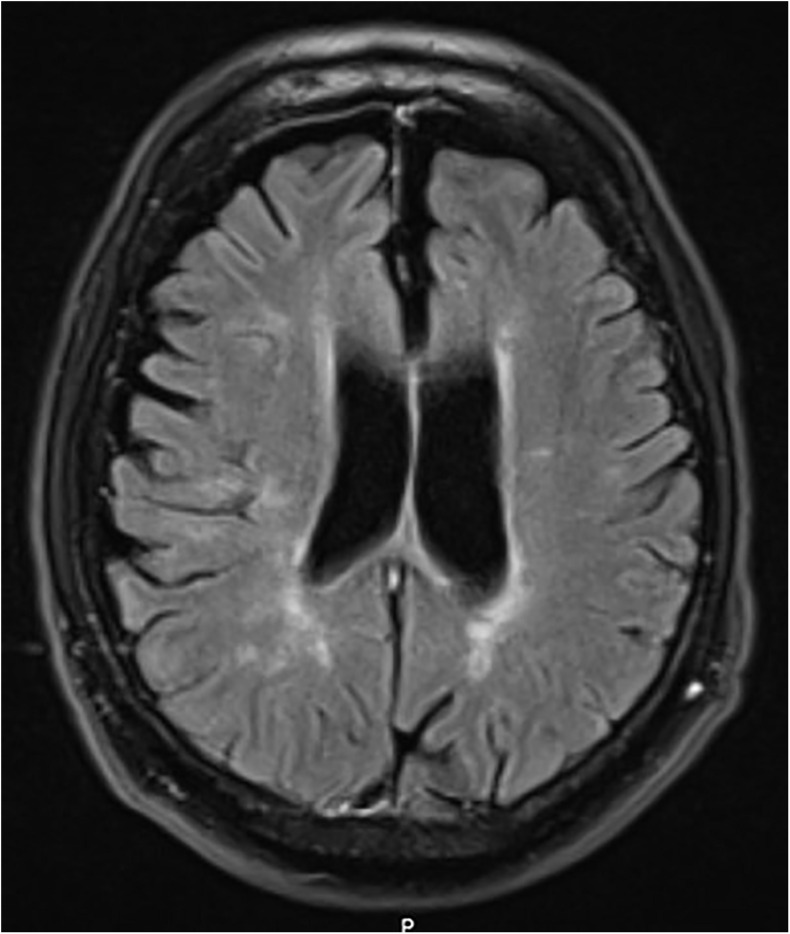
Case 3, LRRK2 mutation carrier, severe MS. MRI at age 50 with white matter lesions and some central and cortical brain atrophy.

Case 6: A 50 years old woman, manager of a small trading company, came to evaluation together with her mother, who, like the grandmother, had been diagnosed with PD. A genetic test showed that they both were carriers of the *LRRK2* G2019S mutation. A PET-scan performed the same year revealed marked basal ganglia pathology (Figure 4). She has now been followed annually for 15 years. She gradually developed moderate bradykinesia and rigidity without any other parkinsonian symptoms and she has not converted to PD. The same year as she had her first PET-scan she started to complain of finger stiffness and swollen, painful joints. She was positive for multiple inflammatory markers and were diagnosed with sero-positive rheumatoid arthritis. She has been on methotrexate therapy for more than 10 years, in combination with other anti-inflammatory drugs. She had to stop working at the age of 65, mainly due to her rheumatism and because of the general COVID-19 situation.

### Non-inflammatory Diseases

#### Cancer

It has been claimed that LRRK2 PD cases have higher prevalence of cancer compared to sporadic PD (Agalliu et al., 2015). This has also been studied in our local Norwegian PD population. We obtained our data from the national Cancer Registry of Norway and we calculated data and cancer outcomes from 857 sporadic PD patients and 76 LRRK2 mutation carriers. The PD population also included 27 LRRK2 PD cases. These were compared data obtained from the national Cancer Registry of Norway and included cancer type and age at cancer onset. All participants were ethnic Norwegians. The LRRK2 mutation carriers had increased risk of non-skin cancer compared with sporadic PD subjects (OR 2.09; 95% CI 1.16–3.77; *p* = 0.015). A significant association was found between the mutation and breast cancer in women (OR 4.58; 95% CI 1.45–14.51; *p* = 0.010). There were no other associations between specific cancer types and the *LRRK2* mutation. There was one otherwise healthy LRRK2 mutation carrier who had been threated since age 50 years for hairy cell leukemia. He was still without signs of PD at the end of the 15 years follow-up period. It was concluded that being a LRRK2 mutation carriers included an increased risk of non-skin cancer compared with sporadic PD subjects. The increased risk for cancer among LRRK2 carriers was mainly driven by the association between harboring the mutation and breast cancer, observed in women (Waro and Aasly, 2018).

### Longitudinal Clinical Evaluations

#### Olfaction

Olfaction was tested in PD patients and in healthy LRRK2 mutation carriers using the UPSIT and B-sit tests. The cohort of LRRK2 carriers and PD patients in central Norway showed the same level of impaired olfactory identification as reported from other centers (Marras et al., 2011; Saunders-Pullman et al., 2011; Sierra et al., 2013; Gaig et al., 2014). The impairment seen in our LRRK2 group was significant although less than in subjects with idiopathic Parkinson disease (Johansen et al., 2014). Others have shown that olfactory dysfunction in LRRK2 patients is positively correlated with reduced uptake of (123)I-meta-iodobenzylguanidine (MIBG) on cardiac scintigraphy, a measure of postganglionic sympathetic cardiac innervation (Valldeoriola et al., 2011).

### Cognition

#### Prevalence of Dementia With Lewy Bodies, DLB

All LRRK2 mutation carriers below the age of 60 years had normal cognitive functions, PD patients included. About half of the LRRK2 PD patients developed cognitive decline as the disease progressed. These cases have been reported in previous publications and at autopsy there was a significant association between cognitive impairment/dementia and the presence of Lewy bodies after adjustment for the degree of Alzheimer disease–related pathology (Kalia et al., 2015).

Previous studies have aimed to determine the risk for conversion to PD in LRRK2 mutation carriers. Norwegian LRRK2 mutation carriers seem to have a significant higher age at conversion compared to individuals carrying the same mutation in Tunisia and Israel (Hentati et al., 2014; Trinh et al., 2014). There have been no studies on the prevalence of DLB in asymptomatic LRRK2 mutation carriers. In our rural districts with scattered population, patients with gradual cognitive decline without the combination of obvious movement disorder signs, often ends up in nursing homes and do not undergo further specific diagnostic procedures. There has been less focus on cognitive decline in LRRK2 mutation carriers without motor signs and who do not convert to PD. Patients who gradually develop cognitive decline often desist from long-time follow-up programs and must be retrieved by active calls from the hospitals. Three of our mutation carriers were located to nursing homes and all had an unspecified diagnose of dementia. None had tremor and a neurological examination showed that they all were rigid and bradykinetic thus fulfilling the criteria for DLB (McKeith et al., 2020). These three cases illustrate the problem of ignoring DLB cases as part of the phenoconversion to PD or to DLB. The three mutation carriers in our cohort had all been diagnosed with unspecified dementia by their local physicians. Whether a LRRK2 mutation carrier converts to PD or DLB is equal from a medical point of view although the histopathological distribution of Lewy bodies in the brain may have slightly different patterns.

#### LRRK2 Mutations Combined With GBA-Mutations

There are 6–7 LRRK2 mutations which are strongly correlated with PD. Other strong PD risk factors are mutations in the gene for Gaucher disease, GBA-mutations. Recently 10 of our patients also had their *GBA* genes fully sequenced and four cases were shown to carry GBA mutations in addition to the G2019S mutation. One 58 years old asymptomatic woman had two GBA mutations. Only one of our cases had converted to PD, at the age of 47 years. The combination *LRRK2* and *GBA* mutations does not seem to have an additive effect to the phenotype. Some have postulated a possible modifying effect of the G2019S mutation on GBA PD (Omer et al., 2020).

### Biomarkers

There have been a large number of studies aiming to find robust biomarkers for PD progression by using LRRK2 pre-clinical cases and compare these to LRRK2 PD, sporadic PD and normal controls. The Norwegian cohort has been part of many of these studies (Shi et al., 2011; Aasly et al., 2012, 2014; Loeffler et al., 2016; Ichinose et al., 2017, 2018). Most studies have been performed in cerebrospinal fluid, CSF, some in blood and others in urine and in saliva (Stewart et al., 2015; Wang et al., 2017).

Most CSF studies have included known metabolites involved in neurodegeneration, like Aβ1-42, tau, α-synuclein, oxidative stress markers, autophagy-related proteins, pteridines, neurotransmitter metabolites, exosomal LRRK2 protein, RNA species, inflammatory cytokines, mitochondrial DNA (mtDNA), and intermediary metabolites. Better technique and smarter machines later added the possibility of studying pteridines, α-synuclein, mtDNA, 5-hydroxyindolacetic acid, β-D-glucose, lamp2, interleukin-8, and vascular endothelial growth factors. Many of the studies suggested to differentiate LRRK2 PD from sporadic PD patients. It was claimed that 8-hydroxy-2′-deoxyguanosine (8-OHdG), 8-isoprostane (8-ISO), 2-hydroxybutyrate, mtDNA, lamp2, and neopterin may differentiate between healthy LRRK2 carriers and LRRK2 PD subjects; and soluble oligomeric α-synuclein, 8-OHdG, and 8-ISO might differentiate healthy LRRK2 carriers from control subjects (Aasly et al., 2012, 2014; Shi et al., 2012; Podlesniy et al., 2016; Vilas et al., 2016; Loeffler et al., 2017; Wang et al., 2017; Ichinose et al., 2018; Klaver et al., 2018). The high number of analytes in combination with the low numbers of investigations of each analyte, and the small sample sizes, together with methodological differences, has limited the conclusions that can be drawn from these studies. There is so far no useful biomarker that can predict PD phenoconversion; not in sporadic PD and not in monogenic PD types. The validity of the analytes identified in these studies needs to be confirmed in larger studies (Aasly, 2020). So far, no robust biomarker for useful PD-specific progression has been found (Loeffler et al., 2019). Neurofilament light chain (NfL), a neuronal cytoplasmic protein highly expressed in large myelinated axons is a well-known marker in a variety of neurological disorders, including inflammatory, neurodegenerative, traumatic and cerebrovascular diseases but it is rather unspecific (Gaetani et al., 2019).

### Autopsies

Five LRRK2 mutation carriers came to autopsy during the 15-year follow-up period, the results have been presented elsewhere (Kalia et al., 2015; Aasly, 2020). Four had developed PD and one was the mutation carrier with MS, without signs of the PD. The presence of LBs were closely correlated to their cognitive functions. An 85-years-old woman died after 25 years of PD. She had no cognitive defects and no LBs. An 80-years-old man died 20 years after disease onset and in a state of very severe dementia. The autopsy showed diffuse LB disease. A 79-years-old male died 20 years after disease onset. His cognitive function was slightly impaired and the autopsy showed only a few LBs. We concluded that there was a clear correlation with the presence of Lewy bodies in the brain and the intellectual performance (Kalia et al., 2015).

### Imaging Markers

*LRRK2* mutation carriers in the Norwegian LRRK2 cohort have taken part in a number of imaging studies. It was soon shown that asymptomatic mutation carriers may have quite extensive basal ganglia dopaminergic defects with very low UPDRS scores. We did a retrospective evaluation of a cohort of 39 participants who underwent Datscan as part of their follow-up. Our goal was to assess whether a combination of systematic clinical testing and different imaging techniques in familial PD cases could detect subclinical signs in the preclinical and prodromal stages of PD. Our cohort of 39 participants were studied with visual analysis of Datscan imaging to assess patterns of dopaminergic degeneration. They were grouped according to diagnostic criteria suggested by the Movement Disorders Society (MDS) Research Criteria for Prodromal PD (Aasly et al., 2005; Gao et al., 2018).

**FIGURE 5:**
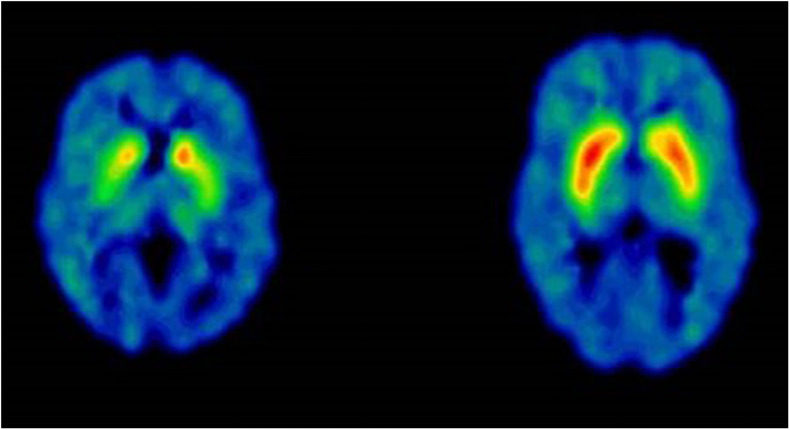
Case 6, 65 years-old healthy LRRK2 mutation carrier with sero-positive rheumatoid arthritis. PET-scan at age 50 DAT ^11^C-MP, methylphenidate. Left scan: Case 6, at age 50, right scan: 54 years-old normal control (with permission from J Stoessl and V Sossi, Pacific Parkinson’s Research Centre, Vancouver, British Columbia, Canada).

The imaging studies showed that LRRK2 mutation carriers above the age of 60 all had some kind of Datscan or PET abnormalities, always reflecting subclinical rigidity and bradykinesia (Ichinose et al., 2018). Corresponding defects has been shown for other neurotransmitters. The Norwegian LRRK2 cohort has taken part in three PET-studies aiming at central dopaminergic, serotoninergic and cholinergic activities. These studies all showed a change in transmitter activity years before conversion to PD (Figure 5). This may be interpreted as an early upregulation for LRRK2-dysfunction or a change of transmitter content in non-neuronal cells (Sossi et al., 2010; Wile et al., 2017; Liu et al., 2018).

## Discussion

This is the first report of the 15 years follow-up of a Norwegian cohort of *LRRK2* G2019S mutation carriers. About one third of the original Norwegian LRRK2 cohort had developed PD after 15 years follow-up. In those who converted to PD the phenotype was close to that seen in sporadic PD and did not differ from patients seen in other *LRRK2* G2019S cohorts. The motor signs were levodopa responsive, they had better olfaction functions than sporadic PD, had less autonomic deficits and they responded very well to DBS. There was an increased prevalence of inflammatory diseases among members of this material. This has not been observed in previous reports from LRRK2 cohorts. Three mutation carriers without PD developed multiple sclerosis, two had sero-positive rheumatoid arthritis and one needed treatment for severe achalasia. This is a relatively high percentage of inflammatory diseases, not commonly seen in combinations with PD.

Multiple sclerosis is rarely seen in PD patients and vice versa. The three MS cases in this report represent *per se* different types of the wide specter of MS. They all fulfilled the criteria for MS and they all had in common that they are *LRRK2* G2019S mutation carriers. The local prevalence of MS among the population in Central Norway is 160 out of 100,000 (Dahl et al., 2004), or 0.16%. The 3% prevalence in a small cohort of LRRK2 carriers could be a coincidence. So far there has been drawn very few connections between PD and MS in genetic studies. In a large Danish register study there was no increase in incidence of PD among MS patients (Nielsen et al., 2014) and reports on both MS and PD are mostly anecdotal case reports (Valkovic et al., 2007). But the association between the immune system and PD needs to be kept in mind. A recent genome-wide association study systemically investigated pleiotropy between PD and autoimmune diseases. There was an overlap between PD and inflammatory diseases, including rheumatoid arthritis and multiple sclerosis, in 17 novel loci, including *LRRK2* (Witoelar et al., 2017). The neuroinflammation in PD may be initiated by activated microglia, upregulated cyclooxygenase-1 and -2-expression, increased inflammatory cytokines and related molecules. In addition, polymorphisms in inflammatory cytokine genes (i.e., tumor necrosis factor-α and interleukin-1β) and cell-surface human leukocyte antigen have been associated with an increased PD risk (Crotty et al., 2020). Recently it was shown that PD patients share a LRRK2 risk variant, N2081D, and a protecting variant, N551K, with Crohn’s disease, CD, patients. This pleiotropic effect of LRRK2 functional variants affect the risk for PD and CD independent of ethnicity (Hui et al., 2018). It is further of interest that CD and MS share common principles for modern treatment. Natalizumab is the most potent drug for MS, mainly by blocking the T lymphocyte intrusion in the central nervous system through the blood-brain barrier, and is effective for CD by blocking cell trafficking into the gut (Pagnini et al., 2017). In both diseases the main effects are achieved through several vascular cell adhesion molecules (Zundler et al., 2019). It is also noted that another very potent drug used for MS, cladribine, is a drug of choice for hairy cell leukemia (Paillassa et al., 2020). Maybe similar treatments should be explored in future LRRK2 PD studies.

The prevalence of rheumatoid arthritis varies between countries and it is highest in high-income western countries. The local prevalence of sero-positive rheumatoid arthritis is 0.35% compared to 2% in this small cohort (Videm et al., 2017). In a survey from Taiwan the cumulative incidence of PD was 2.42% lower in a large RA cohort than in the non-RA cohort (Sung et al., 2016). The lower risk for developing PD in patients affected with RA was not correlated to treatment or use of anti-rheumatic drugs. Other studies have shown that ibuprofen, a non-steroidal anti-inflammatory drug, NSAID, lower the risk for PD while other NSAIDs may not have the same effect (Gao et al., 2011). However, the non-association between treatment and outcome may be differ within PD subgroups. It has been shown that regular NSAID use may be associated with reduced penetrance in LRRK2-associated PD (San Luciano et al., 2020), and that the LRRK2 protein is involved in inflammatory pathways and appears to be modulated by regular anti-inflammatory use. The authors postulate that if LRRK2 set the fire, can non-steroidal anti-inflammatory drug wet the flames? (Crotty et al., 2020).

Achalasia has been connected to PD mainly through case reports. In our LRRK2 case there was also a parallel between start of esophageal symptoms and the clinical manifestation of subclinical parkinsonism. Given its relatively common prevalence (10.82/100,000) achalasia seen in a patient could be a coincidental finding (Sadowski et al., 2010). The etiology of achalasia is unknown but genetic or immune factors may be involved. A number of genes have been shown to increase the risk for achalasia. Polymorphisms of genes for enzymes catalyzing the production of nitric oxide, NO, from L-arginine have been associated with a higher risk for achalasia (Gao et al., 2018). Large genome wide association studies are underway, which may shed further light into genetic predisposition of the disease. Secondly, ample evidence suggests that achalasia is an autoimmune disorder, where an antibody response to a common antigen, perhaps a virus, selectively knocks out esophageal autonomic control mechanisms at the myenteric plexus ganglia and the neuronal level (Villanacci et al., 2010). This theory has been further supported by antibodies that target enteric ganglia which have been identified in the sera of achalasia patients (Moses et al., 2003). Achalasia may be cause by a virus and both Herpes simplex virus type I, measles and human papilloma virus has been suggested as infectious triggers. It has been shown that the nerve plexi/ganglia involved in the motor responses in the distal esophagus show an antigen–antibody response to these agents corresponding to degree of damage contributing greatly to the clinical and smooth muscle findings. This include both the lower esophageal sphincter and esophageal body. Inflammatory responses are mainly seen in type 1 and type 2 with ganglion cells with cell death (Ghoshal et al., 2012).

### Biomarker for LRRK2 PD

Traditional CSF biomarkers in PD patients have not shown any significant change in protein fractions related to neuroimmunological disease mechanisms. It could be more relevant to study small (40–100 nm) extracellular membranous vesicles, exosomes, because they may be carriers of more relevant disease markers which also may include the immune system. Exosomes may been isolated from several body substances like urine, CSF or plasma.

It has been shown that the protein pS1292-LRRK2 protein is robustly expressed in CSF exosomes. In a cohort of Norwegian subjects with and without the G2019S-LRRK2 mutations, with and without PD, we quantified levels of pS1292-LRRK2, total LRRK2, and other exosome proteins in urine from 132 subjects and in CSF from 82 subjects. These results provided insights into the effects of LRRK2 mutations in both the periphery and brain in a well-characterized clinical population and showed that LRRK2 protein in brain exosomes may be much more active than in the periphery in most subjects (Wang et al., 2017). In a similar study plasma-derived extracellular vesicles or exosomes, were isolated from PD, matched healthy controls, and atypical parkinsonism with tauopathies.

Specific groups of markers related to inflammatory and immune cells are located to the surface of exosomes. These markers have been analyzed and correlated to movement disorder patients according to the clinical diagnosis. PD and MSA patients had considerably larger parts of immune markers, indicating that neuroimmune regulation in PD and MSA is different from that observed in atypical parkinsonism. The true positive rate for compound exosome markers showed optimal diagnostic performance for PD. The exome marker curves for PD and MSA were rather congruent and different from those of corticobasal degeneration and progressive supranuclear palsy. In the panels shared by PD and MSA was a transcription factor playing, SP1, which is an important regulator of neuroinflammation in multiple sclerosis (Vacchi et al., 2020).

## Conclusion

The aim of this survey of 15 years follow-up of the original Norwegian LRRK2 cohort was to emphasize the presence of neuroinflammation in a group of LRRK2 mutation carriers. The role of LRRK2 in inflammation and immune system regulation is being increasingly explored. It is expressed in the cells of the innate and adaptive immune system. The *LRRK2* gene is associated with several chronic inflammatory disorders, including Crohn’s disease and leprosy but these results have originated from vast genetic studies like GWAS in heterogenous PD populations. LRRK2 may play a crucial part in the complex interactions of neuroinflammation. The combination of PD and inflammatory diseases is rare. Multiple sclerosis and rheumatoid arthritis are rarely seen with PD. Achalasia may have been reported in early PD but its significance has been debated. The high percentage of inflammatory cases among LRRK2 carriers could indicate that anti-inflammatory drugs may be recommended in risk populations to reduce inflammation and subsequent neurodegeneration.

## Author Contributions

The author confirms being the sole contributor of this work and has approved it for publication.

## Conflict of Interest

The author declares that the research was conducted in the absence of any commercial or financial relationships that could be construed as a potential conflict of interest.
